# Gag-Pol Transframe Domain p6* Is Essential for HIV-1 Protease-Mediated Virus Maturation

**DOI:** 10.1371/journal.pone.0127974

**Published:** 2015-06-01

**Authors:** Fu-Hsien Yu, Ting-An Chou, Wei-Hao Liao, Kuo-Jung Huang, Chin-Tien Wang

**Affiliations:** 1 Department of Medical Research, Taipei Veterans General Hospital, Taipei, Taiwan; 2 Institute of Clinical Medicine, National Yang-Ming University School of Medicine, Taipei, Taiwan; 3 Institute of Public Health, National Yang-Ming University School of Medicine, Taipei, Taiwan; Johns Hopkins School of Public Health, UNITED STATES

## Abstract

HIV-1 protease (PR) is encoded by *pol*, which is initially translated as a Pr160^*gag-pol*^ polyprotein by a ribosomal frameshift event. Within Gag-Pol, truncated p6gag is replaced by a transframe domain (referred to as p6* or p6pol) located directly upstream of PR. p6* has been proposed as playing a role in modulating PR activation. Overlapping reading frames between p6* and p6gag present a challenge to researchers using genetic approaches to studying p6* biological functions. To determine the role of p6* in PR activation without affecting the gag reading frame, we constructed a series of Gag/Gag-Pol expression vectors by duplicating PR with or without p6* between PR pairs, and observed that PR duplication eliminated virus production due to significant Gag cleavage enhancement. This effect was mitigated when p6* was placed between the two PRs. Further, Gag cleavage enhancement was markedly reduced when either one of the two PRs was mutationally inactivated. Additional reduction in Gag cleavage efficiency was noted following the removal of p6* from between the two PRs. The insertion of a NC domain (wild-type or mutant) directly upstream of PR or p6*PR did not significantly improve Gag processing efficiency. With the exception of those containing p6* directly upstream of an active PR, all constructs were either noninfectious or weakly infectious. Our results suggest that (a) p6* is essential for triggering PR activation, (b) p6* has a role in preventing premature virus processing, and (c) the NC domain within Gag-Pol is not a major determinant of PR activation.

## Introduction

The Gag structural protein of human immunodeficiency virus type 1 (HIV-1) is initially synthesized as polyprotein Pr55gag [1]. During or soon after virus budding, Pr55gag is cleaved by viral protease (PR) into four major products—matrix (MA, p17), capsid (CA. p24), nucleocapsid (NC, p7) and p6—plus p2 and p1 spacer peptides (also referred to as SP1 and SP2, respectively) [1]. HIV-1 PR, reverse transcriptase (RT), and integrase (IN) are essential *pol*-encoded enzymes for virus replication. The 5’ end of *pol* overlaps with the 3’ end of *gag*. During Gag synthesis, translating ribosomes shift to a *pol* reading frame at a frequency of 5%, resulting in Pol being translated as a Gag-Pol polyprotein [2]. Within this Gag-Pol, p1-p6gag is truncated and replaced by a transframe domain known as p6* or p6pol.

During Pr55gag virion assembly, Gag-Pol is packaged into virions via interaction with Pr55gag [3,4,5,6,7,8]. It has been suggested that Gag-Pol dimerization triggers PR activation, with activated PR cleaving from Gag-Pol to mediate virus particle maturation via the proteolytic processing of Gag and Gag-Pol. The PR-mediated virus maturation process is necessary for the acquisition of virus infectivity [9,10,11,12,13]. Maintaining Gag-Pol/Gag expression at an approximate ratio of 1/20 is critical for HIV-1 virus particle assembly; any over-expression of PR or Gag-Pol can block virus assembly due to premature Pr55gag cleavage [14,15,16,17,18,19,20].

The Gag-Pol dimerization prerequisite for embedded PR activation implies the involvement of sequences upstream or downstream of PR. We previously reported that a single RT amino acid substitution results in a significant reduction in virus yield in a manner that is similar to treatment with efavirenz [21,22], which is known to enhance RT dimerization [23,24,25]. This suggests that Gag-Pol conformational change or enhanced Gag-Pol dimerization can lead to premature PR activation and premature Pr55gag cleavage. It remains unclear how premature PR activation is blocked in response to Gag-Pol dimerization during virus assembly. p6*, which is located immediately upstream of PR, is thought to play a role in the modulation of PR activation.

There is evidence suggesting that fully functional PR requires p6* removal [26,27,28,29]. Results from in vitro studies suggest that recombinant p6* peptides inhibit PR activity [30,31], with one research team reporting that Gag-PR with a p6* deletion results in significant improvement in Gag-PR proteolytic processing [27]. In addition, NC (located upstream of p6*) has been described as facilitating PR precursor dimerization, which is associated with improved PR precursor auto-processing [32]. Combined, these data suggest that p6* plays a negative role in PR activation, while NC contributes to the triggering of PR activation. Although a recent study suggests that the p6* sequence is not essential for viral replication [33], we previously co-expressed Gag-Pol with Pr55gag, and found that Gag-Pol lacking p6* was significantly defective in terms of mediating virus maturation, despite no clear deficiency in viral incorporation; this suggests a p6* requirement for PR activation [34].

A major limitation of this type of co-expression system is that Pr55gag particles can bud from cells that lack Gag-Pol co-expression, making it difficult to precisely determine PR-mediated virus processing efficiency. To overcome this limitation and to further determine the role of p6* in PR activation, we engineered Gag/Gag-Pol expression constructs containing duplicate PR or p6*-PR sequences, with one of the two PR domains mutationally inactivated. Our results indicate that (a) PR lacking adjacent upstream p6* is severely defective in terms of mediating virus maturation, and (b) the placement of NC upstream of PR or p6*-PR did not significantly enhance virus maturation efficiency.

## Materials and Methods

### Plasmid construction

The parental plasmid used in this study is derived from HXB_2_C. To place an additional copy of HIV-1 protease (PR) or p6*-PR coding sequence in frame at the PR C-terminus, we first engineered a plasmid cassette designated as pBRHIVCla-Sal2548BamHI that contains an HIV-1 coding sequence (from ClaI-nt.831 to SalI-nt.5786) with a created BamHI site at the PR C-terminus. HIV-1 PR-containing cDNA fragments were amplified with a forward primer 5’-CTGTHHATCCTAACTTCCCTCAGGTAACGTTATGGCAA-3’ and a reverse primer 5’-CCTACATACAAGAGCTCCTATTATTGATAGATAAC-3’. The resulting amplified DNA fragments were digested with a combination of BamHI and EcoRV and ligated into pPRCla-Sal2548BamHI, yielding the construct PRII. To construct PRp6*PR, the HIV-1 coding sequence from BglII-nt. 2096 to EcoRV-nt. 2979 was ligated into pPRCla-Sal2548BamHI digested with BamHI anc EcoRV. By similar approaches, constructs PRp6*D, PRD, Dp6*PR and DPR were generated by recombining pBRCla-Sal2548BamHI with a PR-defective version of pBRCla-Sal2548BamHI that contains a PR-inactivated mutation D25 [20].

To construct PRWzPR and PRKzPR, wt and mutant leucine zipper domain of human CREB [35] was PCR-amplified using a forward primer 5’-AATGATGCAGAGAGGCAAT-3’ and a reverse primer 5’-AATGGATCCGATTTGTGGCAGTA-3’. PRWz and PRKz served as templates [36]. The amplified fragments were digested with BamHI and ligated into PRII. To clone NCPR or NC15APR, an XhoI site was created at the PR N-terminus of pBRCla-Sal/2548BamHI by PCR-mediated overlap extension method using an XhoI-containing primer primer 5’-AACTTCCCTCGAGTCACTCTTTGG-3’. The resultant construct was designated pBRCla-Sal/2254/XhoI-2548/BamHI. HIV-1 NC fragments were amplified using primers 5’-AATTCAGCTACTCGAGTGATGCAG-3’ (forward) and 5’-GATCTTCGGATCCAAATTAGCCTG-3’ (reverse), HIVgpt wt or a mutant NC15A that has 15 NC-basic residues replaced with alanines served as templates [36]. PCR-amplified wt or mutant NC fragments then were digested with XhoI and BamHI, and cloned into pBRCla-Sal/2254/XhoI-2548/BamHI. The resultant constructs were digested with ClaI and BamHI and ligated into to PRII and PRp6*PR, yielding the constructs NCPR, NC15APR, NCp6*PR and NC15Ap6*PR. The backbone of all expression constructs is the HIV-1 proviral plasmid HIVgpt [37].

### Cell culture, transfection, and infection

293T cells and HeLa cells were maintained in DMEM supplemented with 10% fetal calf serum. Confluent 293T cells were trypsinized, split 1:10, and seeded onto 10 cm dish plates 24 h before transfection. For each construct, 293T cells were transfected with 20 μg of plasmid DNA by the calcium phosphate precipitation method, with the addition of 50 μm chloroquine to enhance transfection efficiency. For infection, 10 μg of wt or mutant HIVgpt were cotransfected with 5 μg of the VSV-G protein expression vector pHCMV-G [38]. At 48 h post-transfection, virus-containing supernatant was collected, filtered, and mixed with 4 μg/ml polybrene to infect HeLa cells. After 16–18 h, cells were trypsinized, split into dishes and refed with medium containing drug selection cocktail [39]. Selected mycophenolic acid-resistant colonies were fixed and stained with 50% methanol containing 0.5% methylene blue. Numbers of drug-resistant colonies were converted into titers (cfu/ml). Infectivity was expressed as the ratio of the mutant titer to the titer of wt in parallel experiments.

### Western immunoblot analysis

Culture media from transfected 293T cells were filtered through 0.45 μm pore-size filters prior to centrifugation through 2 ml of 20% sucrose in TSE (10 mM Tris-HCl, pH 7.5, 100 mM NaCl, 1 mM EDTA) containing 0.1 mM phenylmethylsulfonyl fluoride (PMSF) at 4°C for 40 min at 274,000 × *g* (SW41 rotor at 40,000 rpm). Viral pellets were suspended in IPB (20 mM Tris-HCl, pH 7.5, 150 mM NaCl, 1 mM EDTA, 0.1% SDS, 0.5% sodium deoxycholate, 1% Triton X-100, 0.02% sodium azide) containing 0.1 mM PMSF. Cells were rinsed with ice-cold phosphate-buffered saline (PBS), scraped from each plate, collected in 1 ml PBS, and pelleted at 2,500 rpm for 5 min. These pellets were resuspended in 250 μl IPB containing 0.1 mM PMSF and subjected to microcentrifugation at 4°C for 15 min at 13,700 × *g* to remove cell debris. Supernatant and cell samples were mixed with equal volumes of 2× sample buffer (12.5 mM Tris-HCl, pH 6.8, 2% SDS, 20% glycerol, 0.25% bromophenol blue) containing β-mercaptoethanol (5%) and boiled for 5 min.

Samples were subjected to SDS-PAGE and electroblotted onto nitrocellulose membranes (blocked with 5% (w/v) non-fat dry milk in Tris-buffered saline containing 0.05% Tween 20 [TBST]) followed by incubation with the primary antibody in TBST buffer containing 5% non-fat dry milk for one hour on a rocking platform at room temperature. Next, membranes were washed three times for 10 min each with TBST and rocked for 30 min with the secondary antibody in TBST buffer containing 5% non-fat dry milk. Blots were again washed three times in TBST for 10 min each. Membrane-bound antibody-conjugated enzyme activity was detected using an enhanced chemiluminescence (ECL) detection system. We used an anti-p24^*gag*^ monoclonal antibody (mouse hybridoma clone 183-H12-5C) at a dilution of 1:5,000 from ascites to detect HIV Gag proteins. Cellular β-actin was detected using a mouse anti-β-actin monoclonal antibody (Sigma), also at a 1:5,000 dilution. Membrane-bound HIV-1 PR was detected with a sheep antiserum. Primary antibody for HIV-1 RT was rabbit antiserum or a mouse anti-RT monoclonal antibody [40,41]. The secondary antibody was either a rabbit anti-mouse, donkey anti-rabbit or anti-sheep (HRP)-conjugated antibody at 1:10,000 or 1:5,000 dilution. Manufacturer’s protocols were followed for HRP activity detection (Pierce).

## Results

### p6* placement between duplicate PR domains mitigated Gag cleavage enhancement

**Fig 1:**
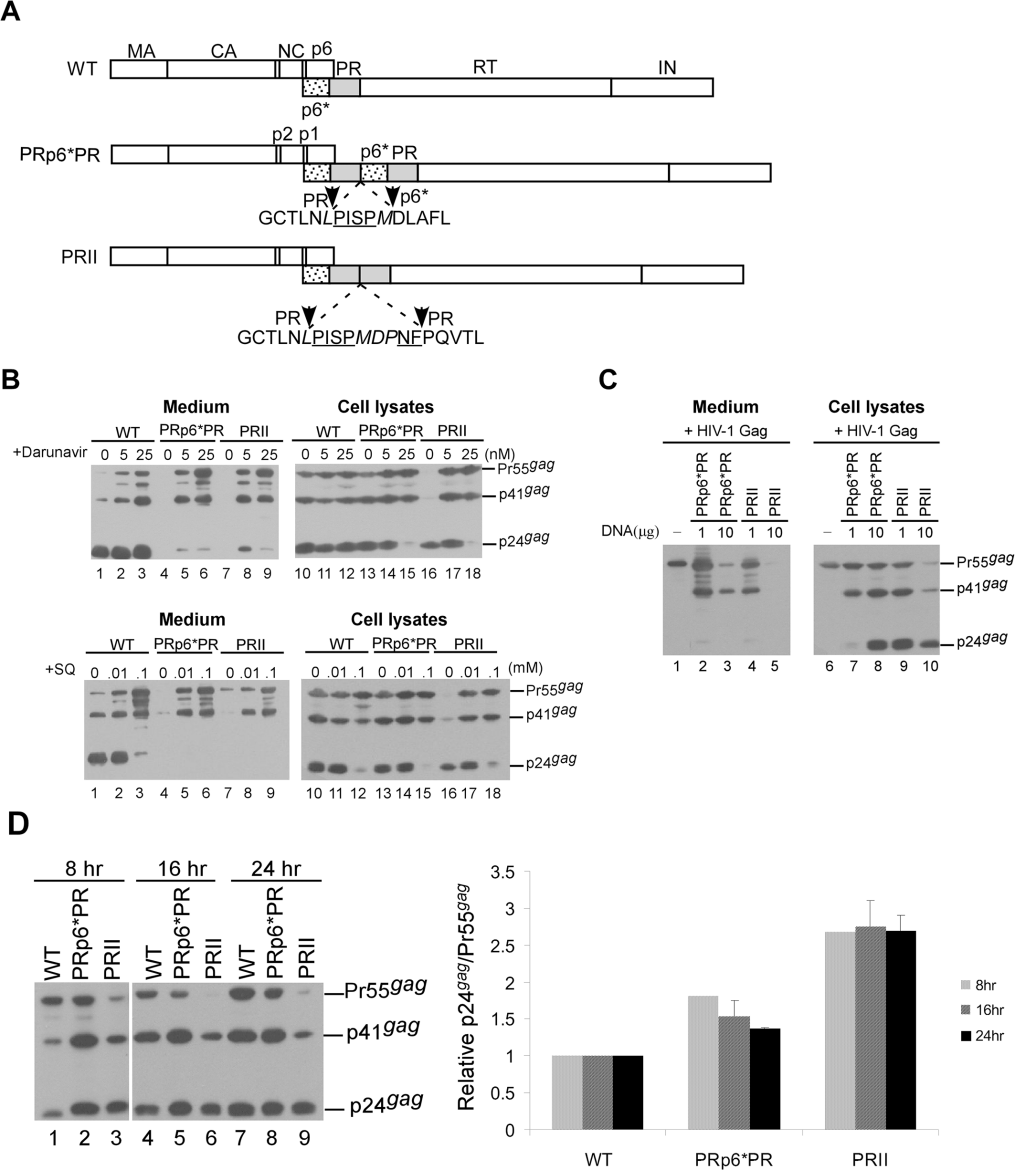
Assembly and processing of HIV-1 mutants containing duplicate PR or p6*PR domains. (A) Schematic representations of wild-type (WT) and recombinant HIV-1 mutants. Indicated are the HIV-1 Gag protein domains MA (matrix), CA (capsid), NC (nucleocapsid), p6, *pol*-encoded p6*, PR, RT, and IN (p31). Underlined “PISP” and “NF” indicate remaining N-terminal RT and C-terminal p6* residues, respectively. Altered or additional residues are in italics. Note the deletion of four N-terminal residues from the second p6* copy. (B) 293T cells were transfected with designated constructs. At 4 h post-transfection, equal amounts of cells were plated on three dishes and either left untreated or treated with a HIV-1 protease inhibitor, either Darunavier or Saquinavir (SQ), at the indicated concentrations. Supernatants and cells were collected 48–72 h post-transfection, prepared, and subjected to Western immunoblotting. (C) Trans-dominant inhibition of HIV-1 Gag particle production. Indicated amounts of PRII or PRp6*PR plasmids were co-expressed with 10 μg of an HIV-1 Gag particle-producing expression vector. At 48 h post-transfection, cells and supernatants were once again collected and analyzed by Western immunoblotting. (D) Time course analysis of wt and mutant Gag processing. 293T cells were transfected with 10 μg of designated constructs. At 4 h post-transfection, equal amounts of cells were placed on three dish plates. Cells were collected 8, 16 and 24 h post-transfection and subjected to Western immunoblotting. Cellular Pr55^*gag*^ and p24^*gag*^ levels were quantified by scanning immunoblot band densities. Ratios of p24^*gag*^ to p55^*gag*^ were determined for each mutant and normalized to those of the wt in parallel experiments. Bars indicate standard deviation.

To determine the effects, if any, of p6* on Gag cleavage efficiency, we constructed multiple HIV-1 Gag/Gag-Pol expression vectors with or without p6* inserted between duplicate PR pairs. These constructs allowed for Gag/Gag-Pol expression from a single plasmid without disrupting the Gag-Pol ribosomal frameshift signal; the resulting constructs were transiently expressed in 293T cells (Fig 1A). Initial Western blot results indicate barely detectable Gag products in culture supernatants following the transient expression of PRII or PR-p6*-PR in 293T cells, likely due to enhanced Gag cleavage from PR over-expression. To test this idea, we collected supernatants 24 h post-treatment with an HIV-1 PR inhibitor (PRI), either saquinavir or darunavir. Medium-associated Gag products that were previously barely detectable became readily observable following PRI treatment (Fig 1B, lanes 4–9). In the absence of PRI treatment, both wild-type (wt) and PRp6*PR transfectants expressed readily detectable Pr55gag, p41gag and p24gag (Fig 1B, lanes 10 and 13); in contrast, Pr55gag and p41gag were barely detectable in PRII transfectants, with mature p24gag representing the primary Gag product (lanes 16). These results suggest that PRII enhanced Gag processing more efficiently than PRp6*PR. To find additional evidence in support of this assumption, PRII or PRp6*PR was co-expressed with a HIV-1 Gag particle-producing expression vector. Our reasoning was that PRII would reduce Gag particle production to a greater extent than PRp6*PR if PRII enhanced Gag cleavage more efficiently than PRp6*PR. As expected, both PRp6*PR and PRII significantly reduced Gag particle production when co-expressed with equal amounts of HIV-1 Gag expression plasmid DNA (Fig 1C, lanes 3 and 5). However, PRII exerted a stronger inhibiting effect than PRp6*PR on Gag particle production under the same experimental conditions (Fig 1C, lanes 4–5 vs. lanes 2–3). Pr55gag and p41gag (instead of p24gag) were predominant in the medium samples (Fig 1C, lanes 2–4), suggesting that unprocessed virions are largely immature. To confirm that PRII exerted a stronger Gag cleavage enhancement effect than PRp6*PR, cell samples were collected 8, 16 and 24 h post-transfection. At 8 h post-transfection and throughout the experiment, PRII had higher p24gag/Pr55gag ratios compared to the wt and PRp6*PR (Fig 1D)—that is, PRII expressed higher levels of PR activity compared to PRp6*PR. This finding is compatible with the proposal that p6* suppresses PR activity.

### Gag-Pol precursors of PRII and PRp6*PR were incorporated into Gag particles following PR inactivation

**Fig 2:**
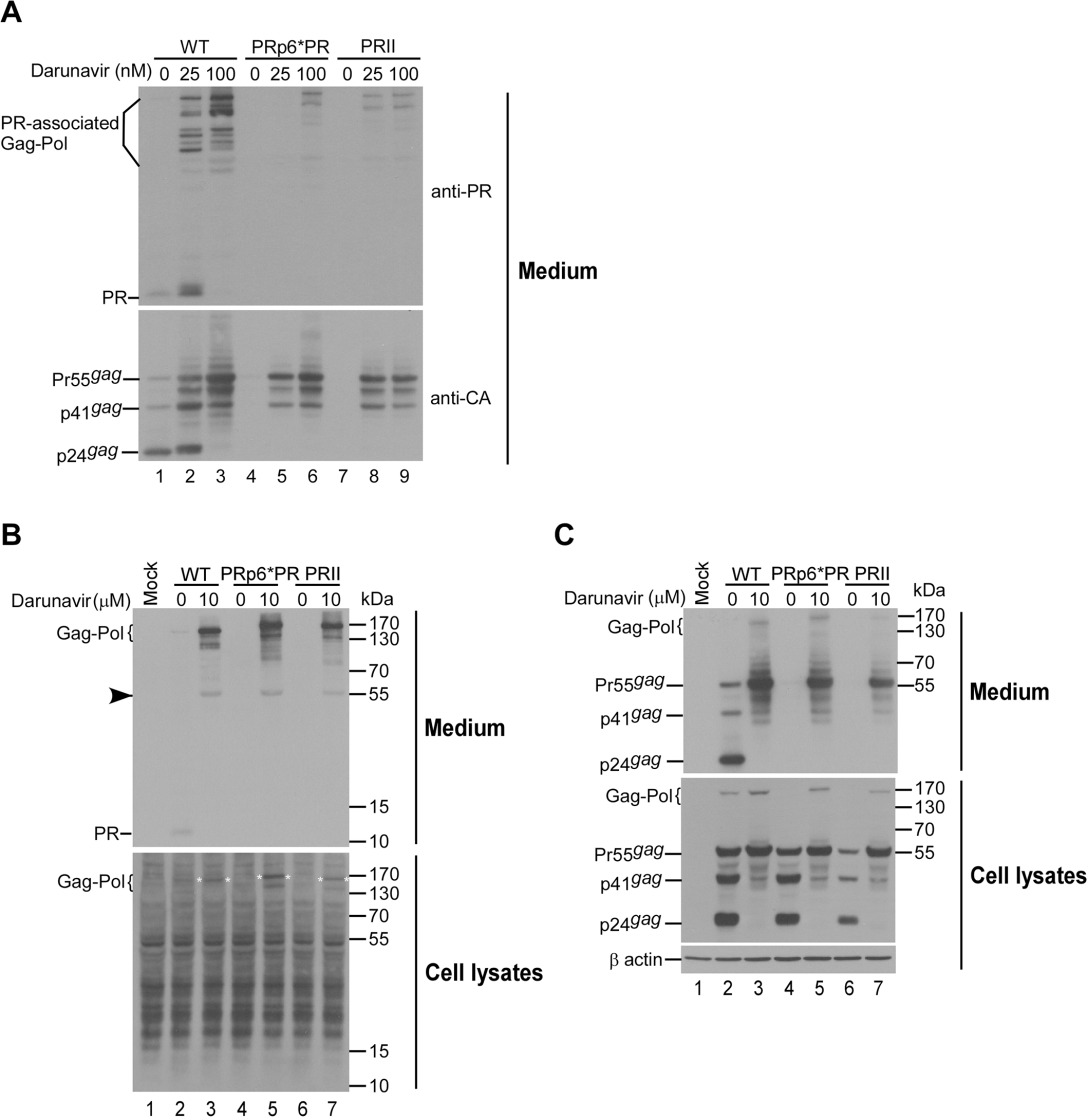
Incorporation of PR and PR-associated Gag-Pol into virus particles. 293T cells were transfected with 10 μg of designated constructs. At 4 h post-transfection, equal amounts of cells were placed on three (panel A) or two (panel B) dish plates, and either left untreated or treated with darunavier at the indicated concentrations. At 48 h post-transfection, cells and supernatants were collected and subjected to Western immunoblotting. PR and PR-associated Gag-Pol were probed with an anti-PR serum (upper panels A and B). Faint bands corresponding to Pr55^*gag*^ likely indicate partial cross-reaction with anti-PR serum (panel B, arrowhead). Membranes were stripped and reprobed with an anti-p24CA monoclonal antibody. Panels B and C are derived from the same blot. Positions of molecular size markers and HIV-1 Gag proteins Pr55, p41 and p24, and PR-associated Gag-Pol proteins are indicated.

All of our attempts to detect PR expression in PRII and PRp6*PR were unsuccessful due to high levels of cellular background signal. We therefore tried to determine PR-associated products in virions following treatment with a PR inhibitor. Since PR activity can affect both virus assembly and Gag-Pol packaging, we adjusted the PR inhibitor dosage to enable the detection of both virions and virion-associated PR-related products, and found that PR was readily detected in wt virions (Fig 2A, lanes 1 and 2). Since fully active PR from PRII and PRp6*PR completely block virus production, we only detected partially cleaved Gag-Pol products in immature virions produced by PRII and PRp6*PR (Fig 2A, lanes 5, 6, 8 and 9). The low virus-associated Gag-Pol levels we observed in PRII and PRp6*PR were likely due to the insufficient suppression of overexpressed PR activity, consequently leading to premature Gag-Pol auto-cleavage prior to packaging into virions. To test this possibility, we assessed virion assembly when PR activity was almost completely blocked by high doses of PR inhibitor. Results indicate that virus-associated PRII and PRp6*PR Gag-Pol levels were comparable to that of the wt when Pr55gag levels in medium were considered (Fig 2B and 2C). Intracellular PR from the wt, PRII or PRp6*PR was barely detectable due to high background signal levels (Fig 2B). Note that PRII and PRp6*PR Gag-Pol were detected when blots were overexposed (Fig 2B, lower panel, asterisks). Combined, the data confirm that both PRII and PRp6*PR are capable of expressing Gag/Gag-Pol and of packaging Gag-Pol into virions when PR activity is blocked.

### Removal of the PR downstream sequence did not exert a major effect on Gag cleavage enhancement due to PR duplication

**Fig 3:**
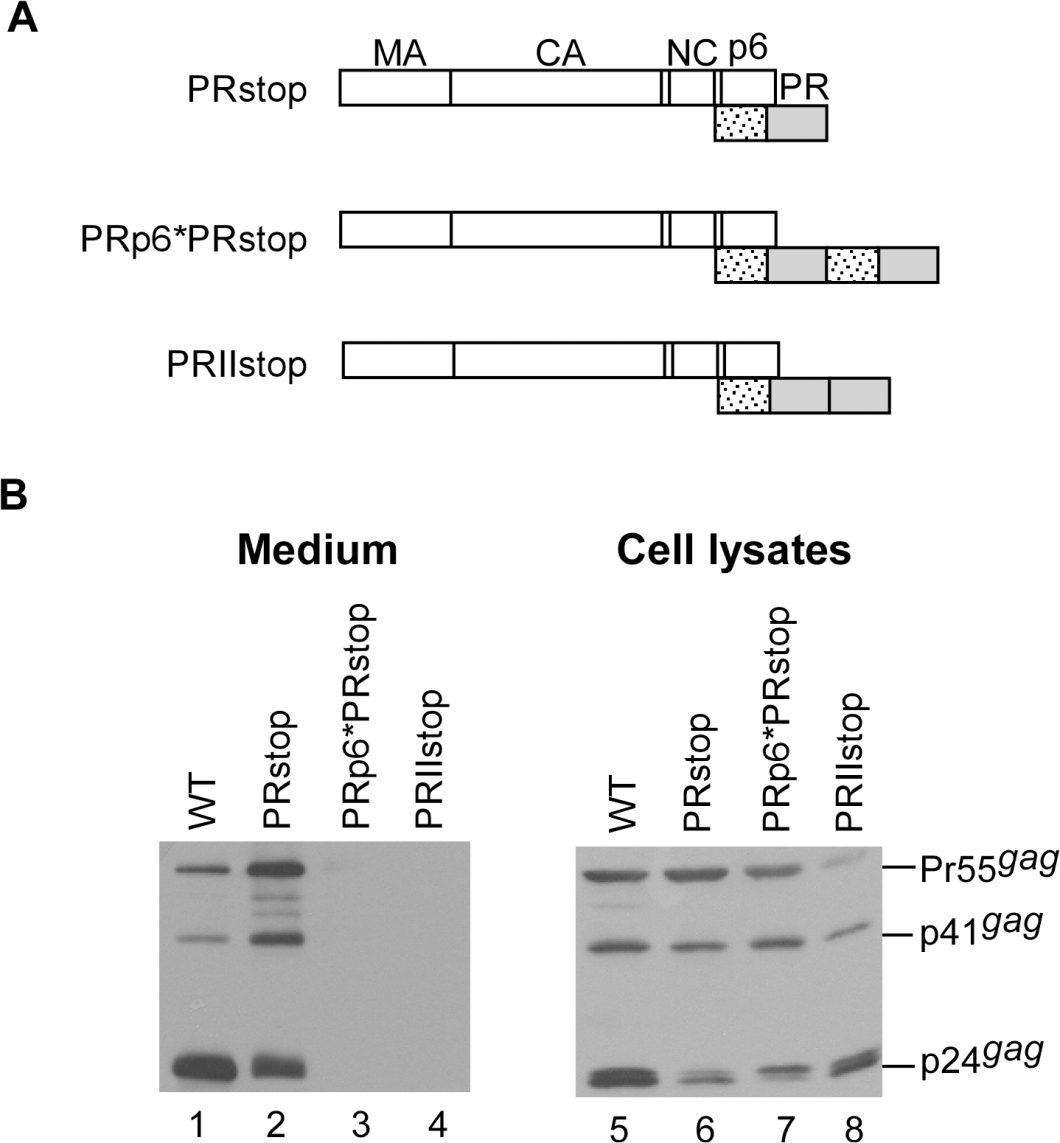
Removal of PR downstream sequences did not significantly impact enhanced Gag cleavage by PR pairs. (A) Schematic representations of HIV-1 Gag and Gag-PR expression constructs. PRstop contained a stop codon insertion at the PR-RT junction (codon sequence 5’TTTCCCATTAGCCCTTAG-3’); RT codons are underlined. Recombining the PRstop with PRp6*PR or PRII (Fig 1) yielded PRp6*PRstop and PRIIstop, respectively. (B) 293T cells were transfected with designated constructs. At 24–48 h post-transfection, cells and supernatants were collected and subjected to Western immunoblotting.

Because PR downstream sequence deletions can significantly reduce Gag cleavage efficiency [42], we expected that barely detectable virus-associated Gag would become more easily detectable if PRII or PRp6*PR Gag cleavage efficiency were significantly reduced following the removal of RT and IN domains. To test this possibility, a stop codon was placed at the PR C-terminus (Fig 3A). Consistent with previously reported results [42], Gag cleavage efficiency was significantly reduced following the deletion of the sequence downstream of PR (Fig 3B, lanes 2 and 6). However, virus-associated Gag was still barely detectable for both PRII and PRp6*PR (Fig 3B, lanes 3 and 4), suggesting that the removal of the RT and IN domains did not significantly affect the Gag cleavage enhancement incurred by PR over-expression. Although some constructs exhibited similar steady-state cellular Gag processing profiles at 48 h post-transfection (PRstop vs. PRp6*PRstop, Fig 3B, lane 6 vs. lane 7; or WT vs. PRp6*PR, Fig 1B, lane 10 vs. lane 13), virion production levels were markedly reduced by the duplicate PRs. We observed that PRIIstop cleaved Gag at a noticeably higher level of efficiency compared to PRp6*PRstop (Fig 3B, lane 8 vs. lane 7), supporting our proposal that p6* prevents premature PR activation and/or premature Gag cleavage.

### PR-inactivated Gag-Pol co-expression reduced overall PR activity

**Fig 4:**
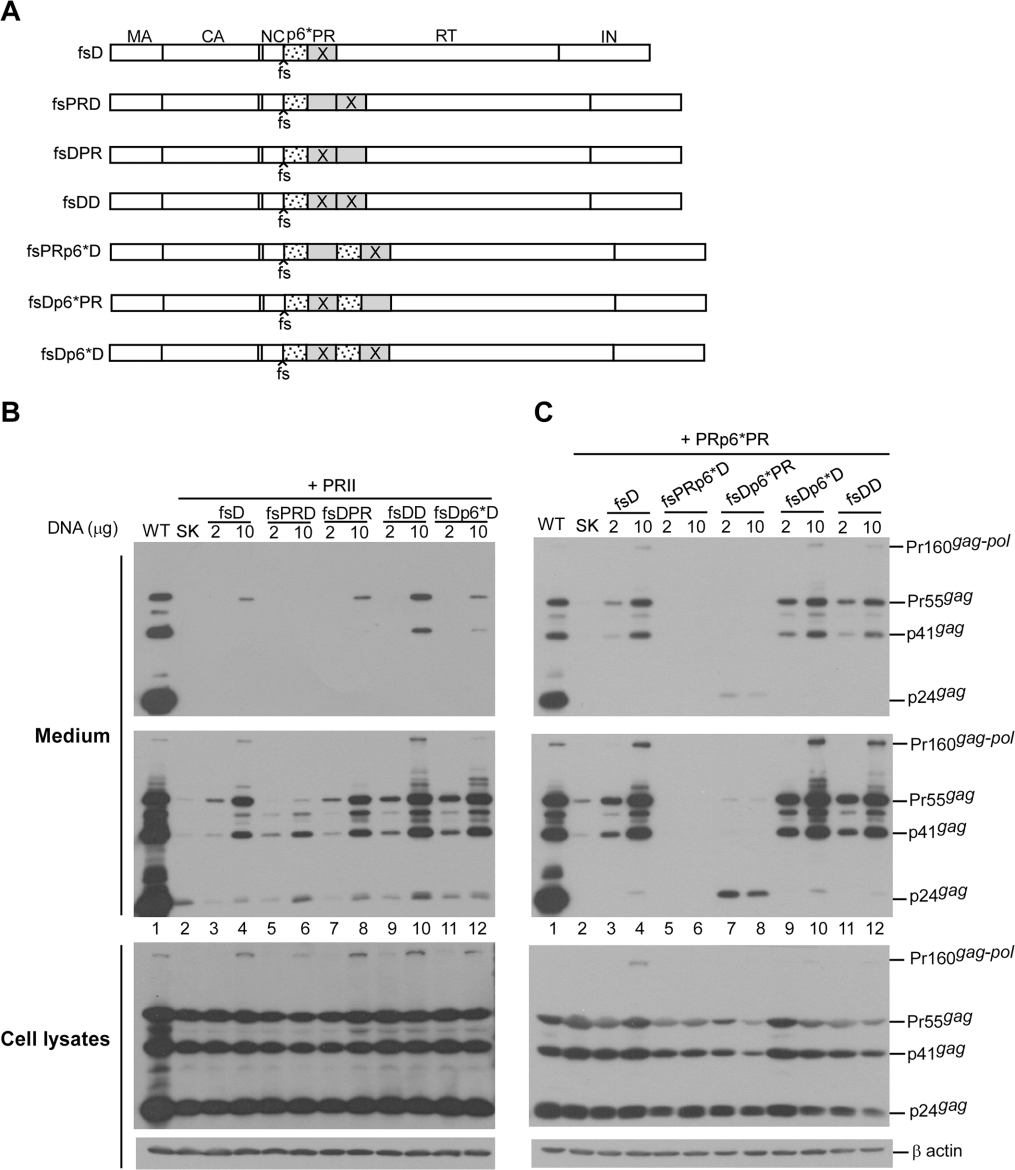
Reduced PRII and PRp6*PR protease activity due to PR-defective Gag-Pol. (A) Schematic representations of HIV-1 Gag-Pol expression constructs. HIV-1 Gag domains, *pol*-encoded p6*, PR, RT and IN are indicated. fs denotes a frameshift mutation that forces *gag* and *pol* into the same reading frame. X denotes a PR-inactivated mutation. (B-C) 293T cells were transfected with 10 μg of PRII or PRp6*PR plasmids, alone or in combination with 2 or 10 μg of one of the designated constructs. Plasmid DNA amounts were maintained at 20 μg by adding pBlueScript SK. At 48–72 h post-transfection, culture supernatants and cells were collected and subjected to Western immunoblotting.

Assuming that PR pair activation is dependent on Gag-Pol dimerization, we posited that Gag-Pol containing inactive PR would interfere with PRII or PRp6*PR protease activity via Gag-Pol/Gag-Pol interaction. To test this idea, PRII and PRp6*PR were co-expressed with their PR-inactivated Gag-Pol versions derived from a Gag-Pol expression plasmid (fsD) containing *gag* and *pol* in the same reading frame. Our results indicate that PRII and PRp6*PR both produced readily detectable virus-associated Gag following co-transfection with equal amounts of PR-inactivated Gag-Pol (fsDD or fsDp6*D) in which both PRs were mutationally inactivated (Fig 4A and 4B, lanes 10 and 12). Since none of the fsD versions produced virus-associated Gag when expressed alone (data not shown), the detected virus-associated Gag was likely derived from the PRII or PRp6*PR due to reduced Gag processing efficiency on the part of the co-expressed fsD versions. These data fit well with observations of PRII and PRp6*PR protease activity suppression by PR inhibitors (Fig 1).

We consistently observed that when PRII was co-expressed with fsDD, it produced virus-associated Gag at higher levels than when it was co-expressed with fsDp6*D. In comparison, when PRp6*PR was co-expressed with fsDp6*D, it produced higher levels of virus-associated Gag than when it was co-expressed with fsDD (Fig 4B and 4C, lane 10 vs. lane 12). This suggests significantly greater reductions in both PRII and PRp6*PR Gag cleavage efficiency due to their PR-inactivated counterparts (fsDD and fsDp6*D, respectively). It is likely that the Gag-Pol of PRII and PRp6*PR interact more efficiently with their PR-inactivated counterparts, resulting in more effective blocking of PR function. Further evidence in support of this assumption is that PRII and PRp6*PR both packaged their respective PR-inactivated Gag-Pol counterparts (fsDD and fsDp6*D) more efficiently than the other types of PR-inactivated Gag-Pol (Fig 4B and 4C, lane 10). We repeatedly observed that PRII produced virus-associated Gag-Pol at lower levels than PRp6*PR when co-expressed with fsD, fsDD or fsDp6*D under the same conditions. This may be due, at least in part, to Gag-Pol being prematurely cleaved by PRII to a greater extent than by PRp6*PR prior to packaging into Gag particles. Interestingly, the co-transfection of PRp6*PR and fsDp6*PR produced detectable virus-associated Gag—primarily p24gag rather than Pr55gag (Fig 4C, lanes 7–8). These data suggest that fsDp6*PR is capable of mediating virus particle maturation in addition to decreasing PRp6*PR-mediated Gag cleavage enhancement.

### Inactivating either one of the two PR domains markedly reduced Gag cleavage efficiency

**Fig 5:**
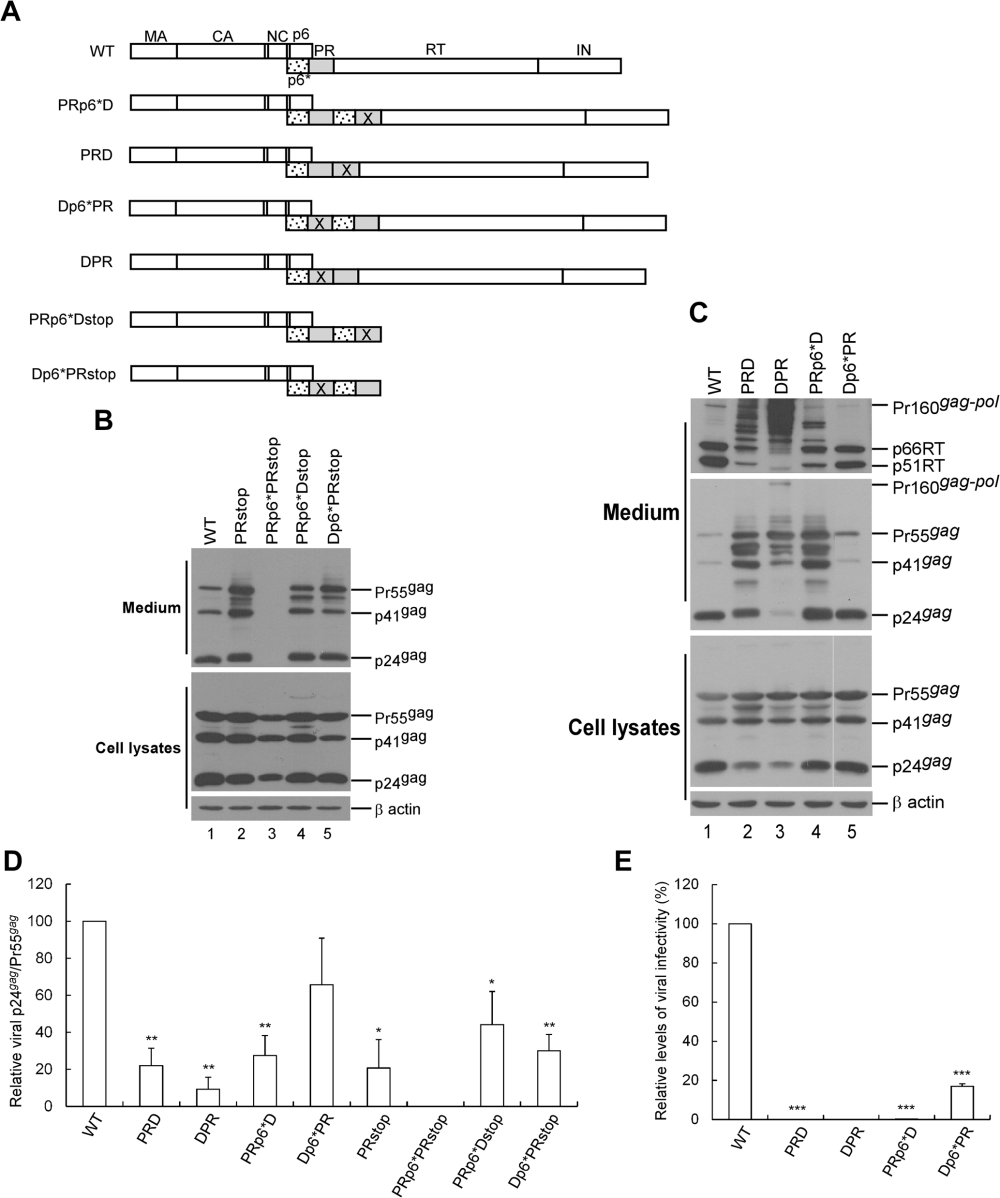
Inactivation of either one of the two PR domains markedly affected virus maturation. (A) Schematic representations of HIV-1 Gag and Gag-Pol expression constructs. X denotes a PR-inactivated mutation. Constructs were derived from PR-inactivated Gag-Pol expression vectors (Fig 4) lacking the gag/pol frameshift mutation. PRp6*Dstop and Dp6*PRstop were derived from recombining the PRstop (Fig 3) with PRp6*D and Dp6*PR, respectively. (B-C) Assembly and processing of HIV-1 mutants. 293T cells were transfected with designated constructs. At 48 h post-transfection, cells and supernatants were collected and analyzed by Western immunoblotting. Equivalent amounts of supernatant samples were probed with anti-p24CA monoclonal antibody or anti-RT antiserum (panel B, top panel). Positions of Pr160gag-pol, RT p66 and p51 subunits, Pr55^*gag*^, p41^*gag*^, and p24^*gag*^ are indicated. (D) Relative virus particle processing efficiency of HIV-1 mutants. Virus-associated Pr55^*gag*^ and p24^*gag*^ levels were quantified by scanning immunoblot band densities as shown in panels B and C. Ratios of p24^*gag*^ to p55^*gag*^ were determined for each mutant and normalized to those of the wt in parallel experiments. Bars indicate standard deviations. *p<0.05; **p<0.01. (E) Relative infectivity of HIV-1 mutants. 293T cells were transfected with the indicated plasmid plus a VSV-G expression vector. At 48 to 72 h post-transfection, approximately 50% of the collected supernatant was subjected to Western immunoblotting. The remaining supernatants were aliquoted and used to infect HeLa cells. Drug-resistant colonies were converted to titers (CFU/ml). Ratios of viral titers to Gag protein levels (obtained via immunoblot band density quantification) were determined for each mutant and normalized to those of the wt in parallel experiments. Mean and standard deviation values for viral infectivity are indicated. ***p<0.001.

Inactivation of both proximal PR (adjacent to Gag) and distal PR (adjacent to RT) resulted in different levels of trans-interference of Gag processing efficiency (Fig 4), implying that the two PRs do not exhibit the same amount of activity. The p6* between the proximal and distal PR domains (hereafter referred to as inter-PR p6*) may serve as a determinant of Gag processing efficiency when one of the two PRs is inactivated. When the proximal and distal PRs within PRII and PRp6*PR were inactivated (Fig 5A), our expectation was that PRII and PRp6*PR would still efficiently mediate Gag cleavage provided that they retained an active PR domain. PRII became noticeably defective in Gag cleavage when one of the two PR domains was inactivated, which agrees with a previous report [16]. Notably, marked impairment of virus particle processing was observed when the proximal PR (DPR) was inactivated (Fig 5B, lanes 2–3 and Fig 5D). Further, inactivation of the distal PR of PRp6*PR impaired virus processing to a greater extent than inactivation of the proximal PR (Fig 5B, lane 4 vs. lane 5).

Substantial amounts of incomplete RT-associated Gag-Pol cleavage products were readily detected in medium (Fig 5B upper panel, lanes 2–4), meaning that the defect in virus processing was more likely due to insufficient PR activation than deficient Gag-Pol incorporation. It is possible that inactivated PR may interfere (either in cis or in trans) with normal PR function when mediating virus particle processing. Normal PR may be susceptible to disturbance by adjacent PR mutants following the removal of inter-PR p6*. In contrast to DPR, which lacked p6* directly upstream of active PR and was severely defective in virion processing, all of the constructs that were capable of producing substantial amounts of virus-associated p24gag contained p6* directly upstream of an active PR (Fig 5B, middle panel). This strongly suggests a p6* requirement for PR activation. Deletions of both RT and IN from Dp6*PR resulted in markedly reduced virus processing efficiency (Fig 5C and 5D), thus confirming the importance of the downstream Pol sequence to PR-mediated virus maturation.

With a virus processing profile similar to that of wt, Dp6*PR is likely capable of producing mature infectious virions. To test this idea, we conducted a single-cycle-infection assay, and found that Dp6*PR exhibited infectivity at a level approximately 20% that of the wt, while the other constructs were either non-infectious or poorly infectious (Fig 5E). Combined, these data suggest that upstream p6* and downstream RT and IN sequences are required for efficient PR-mediated virus processing.

### Adjacent upstream p6* is critical to PR function

**Fig 6:**
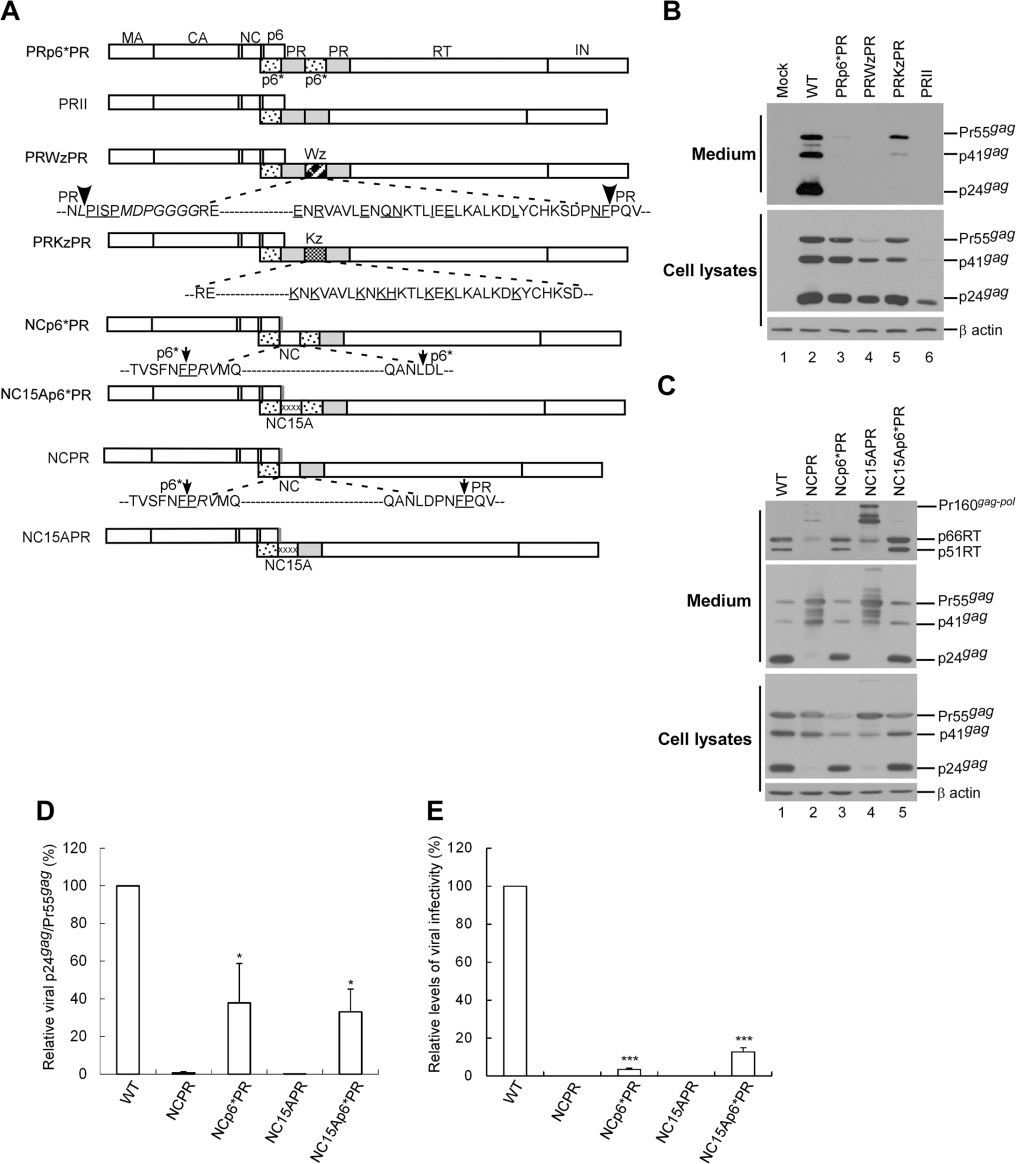
Effects of p6* deletions or substitutions on Gag processing. (A) Schematic representations of HIV-1 Gag and Gag-Pol expression constructs. HIV-1 Gag protein domains and *pol*-encoded proteins are indicated as described in the Fig 1 caption. X denotes a PR-inactivated mutation. “Wz” and “Kz” boxes denote wt and mutant leucine zipper domains, respectively. The mutant LZ contained Lys or His residue replacements for the wt amino acid residues (underlined). The x’s in NC15A denote alanine substitutions of 15 NC-basic residues. Grey vertical lines at the end of p6^*gag*^ denote the deletion of 10 C-terminal residues due o NC replacement for the proximal PR. Amino acid residues in the junction area are indicated. Underlined “PISP” and “NF” indicate remaining N-terminal RT and C-terminal p6* residues, respectively. Residues (F/P) at the p6*/PR cleavage site are underlined. (B-C) 293T cells were transfected with designated constructs. At 48–72 h post-transfection, culture supernatants and cells were collected and subjected to Western immunoblotting. (D) Relative virus particle processing efficiency of HIV-1 mutants. Virus-associated Pr55^*gag*^ and p24^*gag*^ levels were quantified by scanning band densities from immunoblots as shown in panel C. Ratios of p24^*gag*^ to p55^*gag*^ were determined for each mutant and normalized to those of the wt in parallel experiments. Bars indicate standard deviations (*p<0.05). (E) Relative infectivity of HIV-1 mutants. 293T cells were transfected with the indicated plasmid plus a VSV-G expression vector. At 48 to 72 h post-transfection, aliquots of collected supernatants were used to infect HeLa cells or subjected to Western immunoblotting. Viral infectivity was determined by normalizing the ratio of viral titers to Gag protein levels as described in the Fig 5E caption. ***p<0.001.

To find further evidence that the p6* sequence is required for PR activation, a wild-type or mutant leucine-zipper (LZ) motif was substituted for the inter-PR p6* (Fig 6A). The wt LZ (Wz) resulted in barely detectable virus-associated Gag, with cellular Gag cleavage enhanced in a manner similar to that of PRII (Fig 6B, lane 4). This was not a surprising result, since LZ dimerization likely promotes PR activation by facilitating PR precursor dimerization [36]. In contrast, the LZ (Kz) mutant resulted in readily detected virus-associated Gag (Fig 6B, lane 5), suggesting that replacement of inter-PR p6* with a dimerization-defective LZ motif significantly reduced Gag cleavage efficiency. This result supports the hypothesis that the p6* sequence contributes to PR activation.

HIV-1 NC has been proposed as promoting PR precursor dimerization and PR activation in vitro, likely due to the NC capacity for dimerization. To clarify the role of p6* in PR activation and to confirm any NC contribution, we created constructs with NC inserted between the p6* and PR domains (Fig 6A). An NC mutant (NC15A) that did not confer Gag assembly capability served as a control. The results indicate that both NCp6*PR and NC15Ap6*PR (a) displayed Gag processing profiles similar to that of the wt (Fig 6C, lanes 3 and 5), (b) had virus particle processing efficiencies of approximately 30–40% that of the wt (Fig 6D), and (c) were capable of producing infectious virions, although at an infectivity level less than 20% that of the wt (Fig 6E). In contrast, NCPR and NC15APR (both lacking p6* directly upstream of PR) were severely defective in virus processing capability. Both contained incompletely processed (RT-associated) Gag-Pol, similar to results for DPR, PRD and PRp6*D (Fig 5B). The data suggest a defect in Gag-Pol auto-processing, likely due to incomplete PR activation. The data also strongly support the idea that p6* is required for efficient PR activation, and that NC in the Gag-Pol context is not important for PR activation.

## Discussion

Functional HIV-1 PR is dimeric, and the expression of a single-chain HIV-1 PR dimer is sufficient to strongly inhibit virus replication by premature Gag cleavage [16]. We found that Gag/Gag-Pol expression constructs containing tandem PR (PRII) or p6*-PR (PRp6*PR) were capable of blocking virion production by significantly enhancing Gag cleavage. Both PRII and PRp6*PR may experience delayed cleavage at the proximal PR C-terminal following a residue change from F/P to L/P, while still retaining active PRs containing C-terminal extensions [43,44]. If PRII were not capable of releasing PR, or if it triggered a slower release of PR compared to PRp6*PR, then it should have cleaved Gag less efficiently than PRp6*PR, which is not what we observed. Therefore, the presence of altered amino acid residue at the PR cleavage site does not significantly impact our major conclusions.

We repeatedly observed that virus-associated p24gag was readily detected for the wt, but barely detectable in PRII and PRp6*PR medium when treated with low doses of PR inhibitors (Fig 1B, lane 2). We previously reported that a HIV-1 mutant (PRWWz) containing tandem repeat leucine zippers at the HIV-1 PR C-terminus failed to produce virions due to enhanced Gag cleavage efficiency [36]. In that same study we found that the PRWWz also exhibited greater susceptibility to PRI treatment than the wt [36]. Additional research is required to determine why these artificial constructs with Gag processing enhancement are more susceptible to PRI than wt.

Our data indicate that PRII possessed PR capacity at a higher level than PRp6*PR, and that the placement of p6* between the two PR domains resulted in diminished protease activity. This is in agreement with the hypothesis that p6* blocks the premature activation of dimerization proteins or retards PR maturation [45,46,47]. Alternatively (or additionally), when p6* is cleaved from PR, it may block the PR substrate binding cleft, consequently reducing Gag processing efficiency [30].

However, there is also the possibility that PRII forms Gag-Pol or PR dimerization more readily than PRp6*PR, despite reports that p6* has little influence on the dimer formation of wild-type PR [32,48]. Given that the PR-inactivation mutation (D25) does not affect PR dimerization [49], the PR precursor dimerization of PRD or DPR (both lacking inter-PR p6*) may make it easier for defective PR to interfere with wt PR. It is likely that intramolecular PR dimerization is enhanced by the removal of inter-PR p6*. This may explain our finding that PRD and DPR processed virions less efficiently than PRp6*D and Dp6*PR (Fig 5).

We found that the Gag cleavage efficiency of PRp6*PR was noticeably lower when inter-PR p6* was replaced with a dimerization-defective leucine zipper motif (PRKzPR) (Fig 6), and that removal of the inter-PR p6* from Dp6*PR significantly impaired virus particle processing (Fig 5B). Further, the placement of a NC domain directly upstream of PR (NCPR)—which was predicted to support PR activation by promoting PR dimerization—was actually deficient in PR activation (Fig 6C). In contrast, the insertion of p6* between NC and PR conferred a capability to mediate virus maturation. It is not surprising that substantial numbers of virions produced by Dp6*PR, NCp6*PR, and NC15Ap6*PR were noninfectious (despite containing RT and having processing profiles similar to that of the wt), since the addition of extra p6*, inactivated PR, and/or NC sequences to Gag-Pol may have interfered with virus maturation and/or virus replication. Nevertheless, infection assay results strongly suggest a p6* requirement for producing mature infectious virions.

After constructing a HIV-1 provirus plasmid by uncoupling a p6* gene sequence from the p1-p6gag reading frame, Leiherer et al. found that a deletion of 35 amino acids from the 56-amino-acid p6* did not significantly affect virus maturation or infectivity [33], and therefore concluded that the p6* sequence is not essential for viral replication and infectivity. However, they also found that the insertion of a large GFP reporter sequence in the central deleted region of p6* eliminated PR-mediated virus maturation, likely due to the perturbation of PR precursor conformation [33]. Their results suggest that a deletion of 35 residues may not be sufficient for the impairment of p6* function in modulating PR activation.

Zybarth and Carter analyzed the in vitro autoprocessing of a series of Gag-PR polyproteins with progressively larger deletions in the gag coding sequence, and found that deletions involving NC resulted in the loss of PR precursor autoprocessing activity associated with a deficit in PR precursor dimerization [32]. They proposed that NC binding of RNA might facilitate PR dimerization, given the possibility that NC-associated RNA serves as a scaffold facilitating NC-NC interaction and Gag assembly [50,51,52]. However, we failed to find any significant difference in virus particle processing resulting from the insertion of a wt NC or RNA binding-defective NC mutant (NC15A) directly upstream of p6*-PR (Fig 6, NCp6*PR vs. NC15Ap6*PR). According to one previous study, deleting NC from Gag-Pol does not significantly affect Gag-Pol viral incorporation or PR-mediated virus maturation [4]. Combined, these data suggest that NC is not a major determinant in Gag-Pol dimerization or PR activation, which conflicts with Zybarth and Carter’s analysis. A possible explanation is that the constructs assayed by Zybarth and Carter lacked the RT and IN domains, both of which are required for efficient PR activation [42,53]. Accordingly, any contribution of NC to PR activation may be masked or complemented when RT or other Gag domains are present [44,49]. It is possible, but unlikely, that the upstream native NC within NCp6*PR and NC15Ap6*PR makes a significant contribution to triggering PR activation, since NCPR and NC15APR (both of which contain a native NC upstream of native p6*) were found to be severely defective in Gag processing (Fig 6).

During virus assembly, Gag-Pol molecules (which are concentrated at the plasma membrane) tend to trigger PR activation via Gag-Pol/Gag-Pol interactions that may block virus assembly due to premature Gag cleavage. At this point, p6* may serve as a buffer preventing Gag from premature cleavage or PR from early activation. Such a scenario would explain why p6*, when placed between the duplicate PRs, attenuated the activity of over-expressed PR. However, our data suggest that p6* is required for PR activation, in addition to playing a role in preventing premature Gag cleavage or premature PR activation, and that the NC domain within Gag-Pol is not a major determinant of PR activation.
